# CORRELATES OF SUPERAGING IN TWO REPRESENTATIVE SAMPLES OF HISPANIC OLDER ADULTS

**DOI:** 10.1093/geroni/igad104.3250

**Published:** 2023-12-21

**Authors:** Cassidy Doyle, Ross Andel, Joseph Saenz, Michael Crowe

**Affiliations:** University of South Florida, Tampa, Florida, United States; Arizona State University, Tempe, Arizona, United States; Arizona State University, Phoenix, Arizona, United States; University of Alabama at Birmingham, Birmingham, Alabama, United States

## Abstract

‘SuperAgers’ are generally defined as people 80+ years old with episodic memory performance comparable to those 20 years younger. Understanding what distinguishes SuperAgers from those who age normally is valuable for designing population-wide strategies to promote health with aging. Limited knowledge exists to describe characteristics of SuperAgers, and even less is known about Hispanic SuperAgers. We examined indicators of cognitive, physical, and psychological resilience in relation to the likelihood of being a SuperAger. We used data from two population-based studies including Hispanic older adults [Puerto Rican Elder: Health Conditions (PREHCO) study; Health and Retirement Study (HRS)]. SuperAgers were defined as 1) ≥80 years old, 2) recall scores ≥ the median for Hispanic respondents aged 55-64, and 3) no cognitive impairment at any wave (2002-2003, 2006-2007, 2021-2022 in PREHCO; 2002, 2006, 2018 in HRS). In all, 640 PREHCO participants and 180 HRS participants met criteria, of whom 45 (7%) and 31 (17%) met SuperAging criteria. Logistic regressions controlling for age and sex demonstrated that higher education (PREHCO: odds ratio[OR]=1.20, p<.001; HRS: OR=1.14, p=.044) and fewer IADL limitations (PREHCO: OR=0.79, p=.019; HRS: OR=0.58, p=.077), fewer ADL limitations (PREHCO: OR=0.72, p=.031; HRS: OR=0.67, p=.068) (physical resilience), and fewer depressive symptoms (psychological resilience) (PREHCO: OR=0.84, p=.015; HRS: OR=0.69, p=.007) were associated with SuperAging. In addition, number of chronic conditions, self-rated health, or BMI, known indicators of physical health, did not relate to SuperAging, potentially highlighting resilience in aging rather than resistance to aging. These findings highlight potential pathways to promote aging among older Hispanics.

